# Increased Plasma Levels of Thrombin-Cleaved Osteopontin in Patients with Delayed Cerebral Infarction After Aneurysmal Subarachnoid Hemorrhage

**DOI:** 10.3390/ijms26062781

**Published:** 2025-03-19

**Authors:** Kazuaki Aoki, Fumihiro Kawakita, Koichi Hakozaki, Hideki Kanamaru, Reona Asada, Hidenori Suzuki, pSEED Group

**Affiliations:** Department of Neurosurgery, Mie University Graduate School of Medicine, 2-174 Edobashi, Tsu 514-8507, Mie, Japan; kz.mmm.0702@gmail.com (K.A.); fxmx0216@yahoo.co.jp (F.K.); hako.med.xxxx@gmail.com (K.H.); hideki.k.722@gmail.com (H.K.); reona.asd@gmail.com (R.A.)

**Keywords:** delayed cerebral ischemia, matricellular protein, osteopontin, subarachnoid hemorrhage

## Abstract

Osteopontin (OPN), a matricellular protein, is produced as a full-length OPN (FL-OPN) and cleaved by thrombin, thus generating the N-terminal half of OPN (OPN N-half) with new functions. Although plasma FL-OPN levels have been associated with neurovascular events after aneurysmal subarachnoid hemorrhage (SAH), plasma OPN N-half levels have never been investigated. In this study, prospective clinical data and plasma samples were collected from 108 consecutive SAH patients with ruptured aneurysms undergoing acute treatment via surgery, and FL-OPN and OPN N-half levels were measured in plasma with a particular focus on delayed cerebral infarction (DCIn), which has the greatest impact on outcomes. Plasma FL-OPN and OPN N-half levels were intercorrelated and significantly higher in patients with DCIn at days 10–12 post-SAH; a greater area under the receiver-operating characteristic curve was observed for OPN N-half levels, with a cut-off value of 70.42 pmol/L. Multivariate analyses revealed that plasma OPN N-half levels of ≥70.42 pmol/L at days 10–12 were independently associated with DCIn development (adjusted odds ratio, 5.65; 95% confidence interval, 1.68–18.97; *p* = 0.005). Based on the findings of this study and previous reports, an increase in the OPN N-half level may be indicative of a protective mechanism against DCIn development, and, thus, it holds promise as a new therapeutic target against DCIn after aneurysmal SAH.

## 1. Introduction

The prognosis of subarachnoid hemorrhage (SAH), which is caused by a ruptured intracranial aneurysm, remains very poor, with no improvement observed in outcomes [1,2,3]. SAH patients who survive early brain injury suffer secondary disorders such as delayed cerebral ischemia (DCI), and inadequate DCI management can lead to the development of delayed cerebral infarction (DCIn), which is the most influential factor contributing to poor prognosis [4,5]. The molecular mechanism of DCI or DCIn development is complex and not fully understood; however, it is believed to include cerebral artery vasospasm (CVS), cerebral microcirculatory disorders, and neuroelectric disturbance [6,7,8]. Therefore, to develop new therapeutic strategies against DCI or DCIn, it is necessary to identify molecules that can be therapeutic targets.

Osteopontin (OPN) is a 44–75 kDa matricellular secretory glycoprotein and has diverse and multifunctional properties with a broad spectrum of effects, ranging from beneficial to detrimental depending on the biological scenarios of OPN upregulation [9,10]. The OPN gene is translated into a full-length OPN (FL-OPN), which can be proteolytically cleaved by thrombin and transformed into the N-terminal half of OPN (OPN N-half) [9,11]. OPN N-half contains not only highly conserved cell adhesive motifs such as Arg-Gly-Asp (RGD) sequences but also cryptic Ser-Val-Val-Tyr-Gly-Leu-Arg (SVVYGLR)-containing motif sequences, which are exposed only after thrombin cleavage and bind to α4β1, α4β7, and α9β1 integrin receptors, thus exerting a function different from FL-OPN [12,13]. Experimental studies have consistently reported that endogenous FL-OPN is induced in brain tissues after SAH and that the administration of recombinant OPN acts favorably against post-SAH early brain injury and CVS [14,15,16,17,18,19]. In a clinical setting, FL-OPN was secreted into bodily fluids such as peripheral blood after aneurysmal SAH, and the results revealed that increased plasma FL-OPN levels could be used as a predictor or biomarker for post-SAH events and outcomes based on measurement time points: Higher levels were observed at days 1–12 in patients with worse clinical grades at admission and poor 3-month outcomes, at days 4–9 in patients with DCI, at days 1–3 and 10–12 in patients with DCIn, and at days 10–12 in patients with chronic hydrocephalus requiring shunt surgery [20,21,22]. However, to the best of our knowledge, the effects of OPN N-half have never been investigated in relation to SAH. As thrombin has been implicated in post-SAH pathologies [23], OPN N-half can also be generated and may play a role in neurovascular events following SAH. Thus, we hypothesized that circulating OPN N-half levels are also elevated in SAH patients and may be indicative of their neurovascular events, especially DCIn, which is the most decisive and intervening factor contributing to a functional outcome.

The aim of this study was to measure plasma OPN N-half and FL-OPN levels to determine their association with DCIn and the differences between the two in patients with aneurysmal SAH. Although cerebrospinal fluid (CSF) measurements are thought to better reflect intracranial pathology, peripheral blood measurements were examined in this study because they are more amenable to clinical application when used as a biomarker.

## 2. Results

### 2.1. Clinical Variables of Patients With or Without DCIn

**Figure 1:**
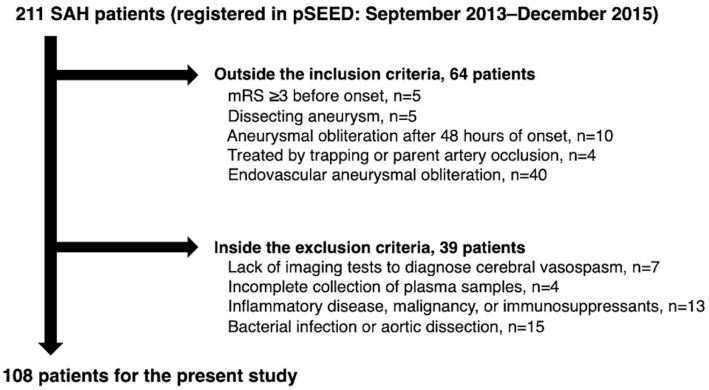
A flowchart of the inclusion and exclusion criteria in this study. mRS, modified Rankin Scale; pSEED, Prospective Registry for Searching Mediators of Neurovascular Events After Aneurysmal Subarachnoid Hemorrhage; SAH, subarachnoid hemorrhage.

Of the 211 consecutive aneurysmal SAH patients enrolled in the Prospective Registry for Searching Mediators of Neurovascular Events After Aneurysmal SAH (pSEED) [24,25,26] during the study period, 108 patients with ruptured saccular aneurysm who were surgically treated within 48 h of SAH onset were analyzed according to the inclusion and exclusion criteria (Figure 1).

The baseline demographic variables are shown in Table 1. The mean age of the 108 patients was 63.6 ± 14.0 years old, and 23 patients (21.3%) were 75 years of age or older. The study population included 76 female patients (70.4%), 98 patients (90.7%) with modified Rankin Scale (mRS) of 0 before onset, 39 patients (36.1%) with preoperative World Federation of Neurological Surgeons (WFNS) grades IV–V, 88 patients (81.5%) with modified Fisher computed tomography (CT) grades 3–4, and 41 patients (38.0%) with acute hydrocephalus. One hundred and two cases of ruptured aneurysm (94.4%) occurred in the anterior circulation, most commonly in the internal carotid artery (39.8%).

The treatment-related and outcome variables are shown in Table 2. CSF drainage was performed in 47 patients (43.5%). Postoperative complications such as cerebral infarction and contusion occurred in 21 patients (19.4%), while angiographic CVS, DCI, and DCIn were observed in 40 (37.0%), 18 (16.7%), and 23 (21.3%) patients, respectively. Endovascular CVS treatment was performed in nine patients (8.3%) and only involved the intra-arterial administration of fasudil hydrochloride. Good outcomes (mRS 0–2 at 3 months post-SAH) were achieved in 64 patients (59.3%).

Considering the baseline demographic and treatment-related variables, a significantly higher number of patients with DCIn had modified Fisher CT grades 3–4 and underwent intra-arterial administration of fasudil hydrochloride, although other factors such as age, WFNS grades, acute hydrocephalus, the site of ruptured aneurysm, postoperative complications, CSF drainage, and prophylactic medications for CVS or DCI were not significantly different between patients with and those without DCIn (Table 1 and Table 2). The results of CT scan and magnetic resonance (MR) imaging analysis revealed that DCIn incidence was also associated with DCI and poor 3-month outcomes, as well as a more frequent occurrence of angiographic CVS, diagnosed via CT angiography (CTA) or digital subtraction angiography (DSA).

### 2.2. Plasma FL-OPN and OPN N-Half Levels

FL-OPN and OPN N-half levels were measured using a commercially available enzyme-linked immunosorbent assay kit in stored plasma samples taken from each patient at four time points: 1–3, 4–6, 7–9, and 10–12 days after SAH. Of the 432 stored samples, seven FL-OPN and 15 OPN N-half plasma samples were considered outliers and excluded from the analysis (for FL-OPN measurements, one sample at days 1–3, two samples at days 4–6, two samples at days 7–9, and two samples at days 10–12 from four patients; for OPN N-half measurements, one sample at days 1–3, three samples at days 4–6, seven samples at days 7–9, and four samples at days 10–12 from eight patients (Supplementary Figure S1)).

**Figure 2:**
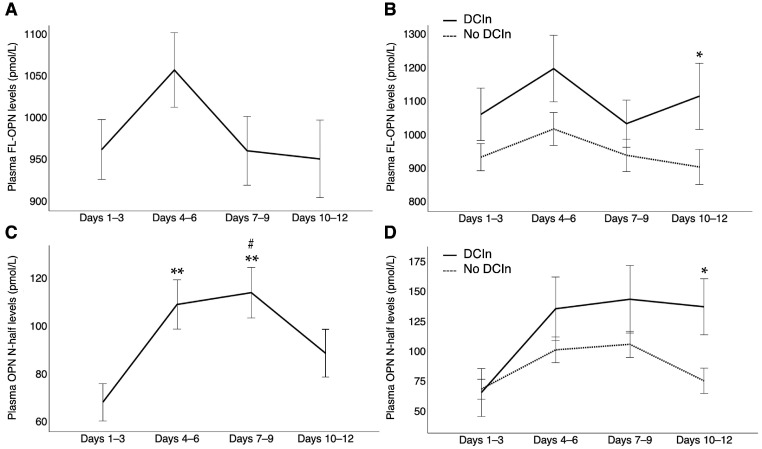
Plasma levels of full-length osteopontin (FL-OPN; **A**,**B**) and N-terminal fragments of osteopontin cleaved by thrombin (OPN N-half; **C**,**D**) observed over time in all patients (**A**,**C**) and separately in patients with (n = 23) and without (n = 85) delayed cerebral infarction (DCIn) after subarachnoid hemorrhage (**B**,**D**). Data are expressed as means ± standard error of the mean; significant differences were observed between the two groups at days 10–12 (* *p* < 0.05, Mann–Whitney U test); ** *p* < 0.05 compared to levels at days 1–3; ^#^ *p* < 0.05 compared to levels at days 10–12 within the group (Mann–Whitney U test).

**Figure 3:**
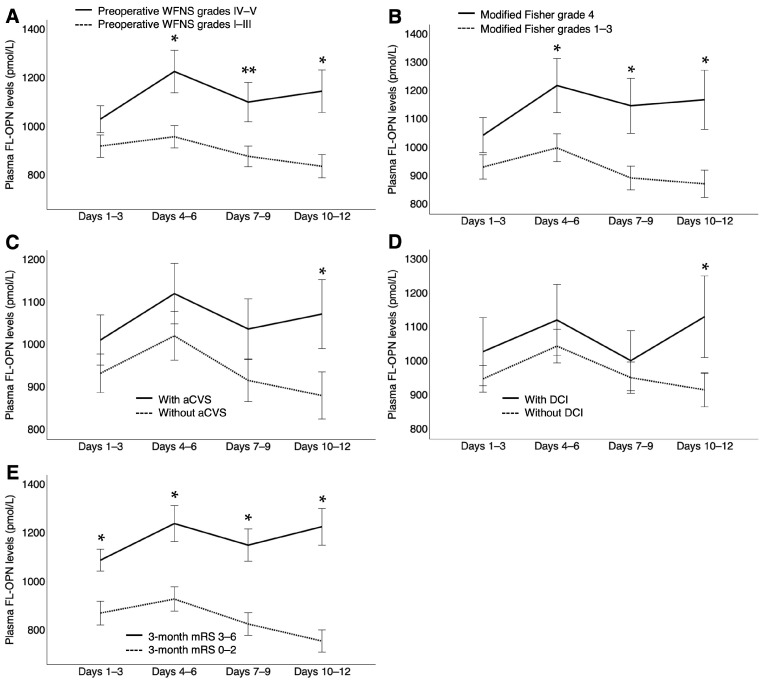
Associations between plasma levels of full-length osteopontin (FL-OPN) at each time point (days 1–3, 4–6, 7–9, and 10–12) and preoperative World Federation of Neurological Surgeons (WFNS) grades (I–III, n = 70, and IV–V, n = 38; **A**), modified Fisher grades (1–3, n = 80, and 4, n = 28; **B**), angiographic cerebral vasospasm (aCVS; presence, n = 40, and absence, n = 68; **C**), delayed cerebral ischemia (DCI; presence, n = 18, and absence, n = 90; **D**), and 3-month modified Rankin Scale (mRS; 0–2, n = 64, and 3–6, n = 44; **E**) after subarachnoid hemorrhage. Data are expressed as means ± standard error of the mean; significant differences were observed between the two groups at each time point (* *p* < 0.05, Mann–Whitney U test; ** *p* < 0.05, unpaired *t*-test).

Plasma FL-OPN levels were significantly increased at all time points after SAH compared with control patients with unruptured intracranial aneurysms (951.56 pmol/L [734.75–1143.86, *p* < 0.001] at days 1–3; 956.96 pmol/L [697.92–1311.26, *p* < 0.001] at days 4–6; 876.09 pmol/L [688.47–1119.15, *p* < 0.001] at days 7–9; and 815.22 pmol/L [638.27–1208.21, *p* = 0.002] at days 10–12 versus 392.62 pmol/L [348.10–621.76] in controls; median [interquartile range, *p* value using Mann−Whitney U test]). After SAH, plasma FL-OPN levels peaked early in the so-called CVS period and then decreased over time (Figure 2A). However, in patients with DCIn, these levels increased again and were significantly higher at days 10–12 (*p* = 0.020) than in those without DCIn (Figure 2B). In patients with DCIn, plasma FL-OPN levels also tended to be higher at days 1–3 (*p* = 0.080) and 4–6 (*p* = 0.061), but the difference was not statistically significant. Patients with preoperative WFNS grades IV–V and modified Fisher CT grade 4 also exhibited significantly higher plasma FL-OPN levels at days 4–6 (*p* = 0.021 and 0.036, respectively), 7–9 (*p* = 0.022 and 0.019, respectively), and 10–12 (*p* = 0.004 and 0.013, respectively), as did those with angiographic CVS and DCI at days 10–12 (*p* = 0.025 and 0.041, respectively) and patients with poor outcomes (3-month mRS 3–6) at all four time points (*p* < 0.001), compared with those without (Figure 3).

**Figure 4:**
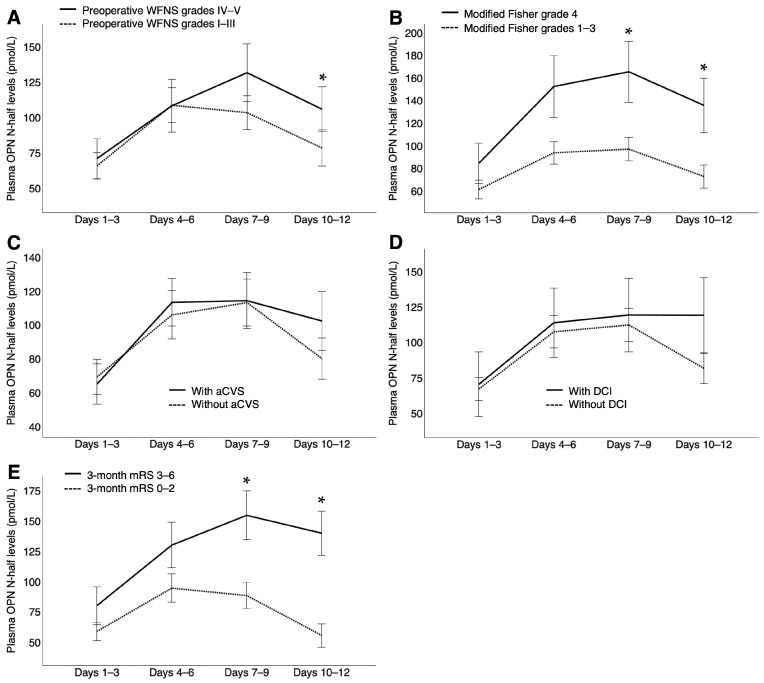
Associations between plasma levels of N-terminal fragments of osteopontin cleaved by thrombin (OPN N-half) at each time point (days 1–3, 4–6, 7–9, and 10–12) and preoperative World Federation of Neurological Surgeons (WFNS) grades (I–III, n = 70, and IV–V, n = 38; **A**), modified Fisher grades (1–3, n = 80, and 4, n = 28; **B**), angiographic cerebral vasospasm (aCVS; presence, n = 40, and absence, n = 68; **C**), delayed cerebral ischemia (DCI; presence, n = 18, and absence, n = 90; **D**), and 3-month modified Rankin Scale (mRS; 0–2, n = 64, and 3–6, n = 44; **E**) after subarachnoid hemorrhage. Data are expressed as means ±standard error of the mean; significant differences were observed between the two groups at each time point (* *p* < 0.05, Mann–Whitney U test).

Plasma OPN N-half levels were also significantly increased at all time points after SAH compared with control patients with unruptured intracranial aneurysms (37.76 pmol/L [15.19–86.92, *p* < 0.001] at days 1–3; 81.77 pmol/L [26.31–131.73, *p* < 0.001] at days 4–6; 80.88 pmol/L [40.25–136.43, *p* < 0.001] at days 7–9; and 48.48 pmol/L [18.03–105.95, *p* < 0.001] at days 10–12 versus 8.57 pmol/L [2.21–9.08] in controls; median [interquartile range, *p* value using Mann−Whitney U test]). After SAH, plasma OPN N-half levels markedly increased from days 1–3 to days 4–6 and then decreased from days 7–9 to days 10–12 (Figure 2C). However, the difference in plasma OPN N-half levels between patients with DCIn and those without DCIn gradually increased, with these levels remaining elevated in those with DCIn without any decline until days 10–12, when the difference reached statistical significance (*p* < 0.001; Figure 2D). Even without excluding outliers, plasma OPN N-half levels were significantly higher in patients with DCIn than in patients without DCIn at days 10–12 after SAH (*p* = 0.001; Supplementary Figure S2). Plasma OPN N-half levels were also significantly higher in patients with preoperative WFNS grades IV–V at days 10–12 (*p* = 0.018) and in those with modified Fisher CT grade 4 and 3-month poor outcomes at days 7–9 (*p* = 0.007 and 0.001, respectively) and 10–12 (*p* = 0.007 and < 0.001, respectively) than in those without, although no significant differences were observed in plasma OPN N-half levels at any sampling point between patients with and those without angiographic CVS, nor between those with and those without DCI (Figure 4).

Regarding gender differences, both plasma FL-OPN and OPN N-half levels tended to be higher in female patients (female:male = 1008.42 ± 463.67:900.83 ± 332.95 pmol/L, *p* = 0.052; and 101.75 ± 105.99:81.50 ± 85.07 pmol/L, *p* = 0.188, respectively; Mann–Whitney U test), which reflected the fact that the proportion of preoperative WFNS grades IV–V was higher in female patients than in male patients (42.1% and 21.9%, respectively; *p* = 0.046, Pearson’s chi-squared test). In the control patients with unruptured aneurysms, no gender differences were observed in plasma FL-OPN or OPN N-half levels (female:male = 469.35 ± 284.94:475.26 ± 308.56 pmol/L, *p* = 0.974; and 5.93 ± 7.65:7.30 ± 3.43 pmol/L, *p* = 0.732, respectively; unpaired *t*-test).

### 2.3. Multivariate Analysis Using DCIn as the Dependent Variable

**Figure 5:**
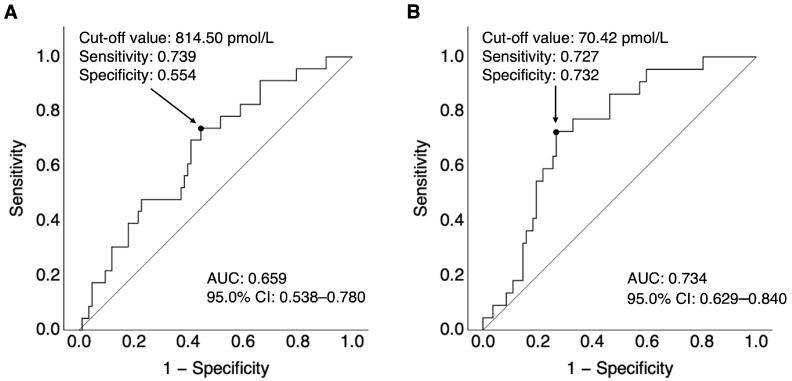
The receiver-operating characteristic curve analysis of plasma levels of full-length osteopontin (**A**) and N-terminal fragments of osteopontin cleaved by thrombin (**B**) at days 10–12 after subarachnoid hemorrhage according to the presence or absence of delayed cerebral infarction. AUC, area under the curve; CI, confidence interval.

To differentiate patients with DCIn from those without DCIn, a receiver-operating characteristic (ROC) curve was plotted for plasma FL-OPN and OPN N-half levels at days 10–12. The area under the curve (AUC) for FL-OPN levels was 0.659, with a cut-off value of ≥814.50 pmol/L using the Youden index, a sensitivity of 73.9%, and a specificity of 55.4% (Figure 5A). Regarding OPN N-half levels, the cut-off value calculated using the Youden index was 70.42 pmol/L (AUC, 0.734; sensitivity, 72.7%; specificity, 73.2%; Figure 5B). As the cut-off of plasma OPN N-half levels was associated with a greater AUC than that of plasma FL-OPN levels, and they were significantly correlated (r = 0.387), plasma OPN N-half levels of ≥70.42 pmol/L at days 10–12 was selected as the candidate variable for subsequent multivariate analysis.

Considering plasma OPN N-half levels of ≥70.42 pmol/L at days 10–12 and the significant variables other than 3-month mRS that were used in univariate analyses, significant correlations were observed between angiographic CVS and DCI (r = 0.583), DCI and intra-arterial administration of fasudil hydrochloride (r = 0.584), and angiographic CVS and intra-arterial administration of fasudil hydrochloride (r = 0.393). Therefore, modified Fisher CT grades 3–4; DCI, which exhibited the smallest *p* values among the intercorrelated clinical variables; and plasma OPN N-half levels of ≥70.42 pmol/L at days 10–12 were used as candidate variables for multivariate analysis to identify the independent factors associated with DCIn incidence. Multivariate analysis revealed that DCI occurrence (adjusted odds ratio [aOR], 10.49; 95% confidence interval [CI], 2.89–38.12; *p* < 0.001) and plasma OPN N-half levels of ≥70.42 pmol/L at days 10–12 (aOR, 5.65; 95% CI, 1.68–18.97; *p* = 0.005) were independent factors contributing to DCIn incidence (Table 3).

## 3. Discussion

In this study, we first measured plasma FL-OPN and OPN N-half levels simultaneously in patients with aneurysmal SAH who underwent treatment using surgical clipping, and the results revealed that (1) plasma OPN N-half levels increased more markedly and exhibited a later peak than plasma FL-OPN levels; (2) both FL-OPN and OPN N-half plasma levels were significantly higher in patients with DCIn than in those without DCIn at days 10–12, and plasma OPN N-half levels were more strongly associated with DCIn incidence than plasma FL-OPN levels; (3) in contrast to plasma FL-OPN levels, significant differences were not observed in plasma OPN N-half levels at days 10–12 between patients with and those without angiographic CVS; and (4) plasma OPN N-half levels of ≥70.42 pmol/L at days 10–12 post-SAH were independently associated with the occurrence of DCIn. Plasma OPN N-half levels increased with delay and were significantly higher in patients with DCIn at days 10–12, when the peak period of DCI occurrence had passed [6], than in patients without DCIn. Furthermore, OPN has been repeatedly reported to have a neuroprotective effect against SAH [9]. Taken together, this study suggests that OPN N-half may also be neuroprotective and that its effects may be stronger than those of FL-OPN. OPN N-half may increase depending on the severity of DCIn after its onset, regardless of angiographic CVS, but researchers believe that naturally produced OPN N-half levels are insufficient to suppress DCIn, the most important treatable prognostic factor [4].

After SAH, FL-OPN is transcriptionally upregulated by growth factors, cytokines, and endothelins following early brain injury and DCIn occurrence, against which it has been reported to exert neuroprotective effects by inhibiting processes such as neuroinflammation, blood–brain barrier disruption, neuronal apoptosis, and CVS by binding to RGD-dependent integrins and CD44 receptors [9,27]. Blood clots in the subarachnoid space typically start dissolving and releasing thrombin, the concentration of which remains high in the CSF at least until 7–9 days post-SAH, although the exact time course of thrombin concentrations in the CSF has not been investigated [23]. The entry of prothrombin into the brain through disruption in the blood–brain barrier and its production by neurons and astrocytes also contribute to high levels of thrombin in the CSF at a later stage after SAH [28,29]. Thrombin not only causes intravascular microthrombosis, neuroinflammation, blood–brain barrier disruption, neuronal apoptosis, and CVS, resulting in DCIn [23], but it also cleaves FL-OPN, thus yielding OPN N-half and leading to the exposure of cryptic SVVYGLR-containing motif sequences [9,12,13]. Thus, although there have been no reports on the time course of OPN N-half concentrations in the plasma after SAH, the findings of this study indicating that these levels were significantly higher in patients with DCIn at days 10–12 are not considered contradictory.

**Figure 6:**
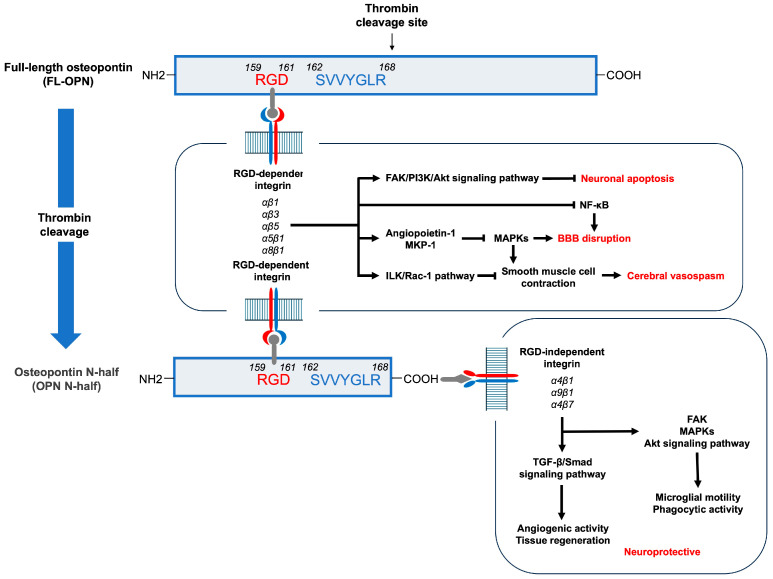
Possible neuroprotective mechanisms of full-length osteopontin (FL-OPN) and osteopontin N-half (OPN N-half). Both FL-OPN and OPN N-half contain an Arg-Gly-Asp (RGD) sequence and bind to RGD-dependent integrins on the cell surface, inducing angiopoietin-1 and mitogen-activated protein kinase (MAPK) phosphatase-1 (MKP-1), inhibiting nuclear factor κB (NF-κB), activating focal adhesion kinase (FAK) to activate phosphatidylinositol 3-kinase (PI3K)–Akt signaling or upregulate autophagy-related proteins, and/or activating the integrin-linked kinase (ILK)–Rac-1 pathway, resulting in suppressing neuronal apoptosis, blood–brain barrier (BBB) disruption, and cerebral vasospasm. Thrombin cleavage of FL-OPN generates the OPN N-half, which exposes a Ser-Val-Val-Tyr-Gly-Leu-Arg (SVVYGLR)-containing motif sequence that activates the transforming growth factor (TGF)-β/Smad signaling pathway to promote tissue regeneration and angiogenesis through binding to the TGF-β receptor or RGD-independent integrins, or enhances microglial motility and phagocytic activity through activation of the FAK, MAPK, and Akt signaling pathways. All mechanisms may act neuroprotectively, and the OPN N-half is thought to be more neuroprotective than FL-OPN because OPN N-half acts through both the RGD- and SVVYGLR-containing motif sequences.

The RGD sequence, a common motif in FL-OPN and OPN N-half, is involved in neuroprotective mechanisms, including anti-inflammatory, antiapoptotic, proangiogenic, and phagocytotic functions [9,30,31,32]. Neuroprotective signaling through RGD-dependent integrins includes the induction of angiopoietin-1 and mitogen-activated protein kinase phosphatase-1, as well as the inhibition of nuclear factor-κB, the activation of focal adhesion kinase to activate the phosphatidylinositol 3-kinase–Akt signaling or to upregulate autophagy-related proteins, and the activation of the integrin-linked kinase–Rac-1 pathway [9]. Experimental studies investigating transient cerebral ischemia have revealed that thrombin cleavage led to a twofold enhancement in the neuroprotective properties of FL-OPN, and the resulting OPN N-half was significantly more neuroprotective than either FL-OPN or the remaining C-terminal half of OPN [33]. This is believed to be due to the increased binding of the OPN N-half generated through thrombin cleavage of FL-OPN to RGD-dependent integrin receptors as well as its access to RGD-independent integrin receptors via the SVVYGLR-containing motif sequence [30,33,34]. Reports on the functions of the SVVYGLR sequences are limited compared with studies on RGD sequences. However, the SVVYGLR motif of OPN N-half has been found to promote tissue regeneration and angiogenic activity by binding to transforming growth factor (TGF)-β receptors or RGD-independent integrins composed of α4β1, α4β7, and α9β1, which are directly involved in the regulation of the activation of the TGF-β/Smad signaling pathway or influence it [34]. In addition, integrin–TGF-β crosstalk has also been implicated in SVVYGLR motif-mediated responses [34]. An in vitro study using a mouse microglia cell line reported that both RGD and SVVYGLR motifs similarly and additively enhanced microglial motility and phagocytic activity through integrin-mediated activation of focal adhesion kinase, mitogen-activated protein kinase, and Akt signaling pathways, which is likely one of the neuroprotective mechanisms [35]. These previous reports, as well as the analysis of the time course of plasma OPN N-half levels in this study, indicate that OPN N-half may exert neuroprotective effects against DCIn after aneurysmal SAH (Figure 6).

In this study, plasma OPN N-half levels were higher in patients with preoperative WFNS grades IV–V, modified Fisher CT grade 4, and 3-month poor outcomes than in those without, which may be attributed to the following factors: (1) DCIn likely occurs in severe cases with preoperative WFNS grades IV–V and modified Fisher CT grade 4, resulting in poor outcomes; (2) plasma OPN N-half may increase at days 10–12, reflecting the severity of precedingly developed DCIn; (3) upregulated OPN N-half may exert a neuroprotective function against DCIn or aid in recovery, but the endogenous OPN N-half levels are likely insufficient to contribute to recovering from DCIn or preventing DCIn from getting worse; and (4) consequently, high plasma levels of OPN N-half may be associated with poor outcomes after SAH. The same phenomena were observed with FL-OPN and another matricellular protein, the pigment epithelium-derived factor, which are known to have neuroprotective properties; however, their plasma levels were higher in patients with ultimately poor outcomes [22,24]. Thus, our hypothesis regarding the neuroprotective properties of OPN N-half may be valid, which requires further investigation. In the future, it may be possible to prevent or treat DCIn by amplifying OPN N-half levels.

This study has some limitations. First, the data were obtained exclusively from patients with ruptured intracranial aneurysms treated via surgical clipping as the treatment modalities differed in invasiveness and, therefore, influenced plasma OPN levels (Supplementary Figure S3). In addition, we excluded patients with any disease or condition that may affect OPN levels. Thus, the generalizability of the findings is limited. Second, whether the OPN N-half levels originated from the intracranial space remains unclear, as we did not measure OPN N-half levels in the CSF. As the outflow of intracranial substances into the bloodstream is affected by lymphatic drainage and the blood–brain barrier, both of which are impaired after SAH [6], plasma levels may not accurately reflect the intracranial conditions. However, Abate et al. [36] reported that an increase in FL-OPN in the plasma was likely of intracranial origin, as plasma and CSF FL-OPN levels followed a similar course, with significantly higher FL-OPN levels observed in the CSF after aneurysmal SAH. Furthermore, previous studies have suggested that the time course of thrombin activation after SAH was similar in the peripheral blood and the CSF [23,37]. Taken together with the previous studies, the findings of this study suggest that plasma OPN N-half levels, resulting from thrombin cleavage of FL-OPN, are possibly indicative of the intracranial upregulation of OPN N-half, although this hypothesis needs to be confirmed in future studies.

To overcome these limitations and make the results more generalizable, clinical studies involving a larger number of patients in which peripheral blood and CSF are collected simultaneously over time are required to simultaneously measure FL-OPN, OPN N-half, and thrombin concentrations and to clarify their relationships with thrombin activation or DCIn. To understand the expression mechanism and function of OPN N-half, it may also be useful to investigate its relationship with neuroinflammation, central nervous system-derived cell death, and recovery-related biomarkers in peripheral blood and CSF, even though they are non-specific [38,39]. In addition, it is necessary to clarify how coil embolization treatment for ruptured cerebral aneurysms and various coexisting diseases affect these measurements. Furthermore, animal experiments using recombinant OPN N-half and neutralizing antibodies against OPN N-half are required to clarify the functional role and mechanism of OPN N-half in SAH and whether it can be a therapeutic target. However, this is the first study to reveal the time course of plasma OPN N-half levels in SAH patients and the strong association between plasma OPN N-half levels and DCIn, justifying further clinical and experimental studies.

## 4. Materials and Methods

All procedures involving human participants were in accordance with the ethical standards of the institutional and/or national research committee and the 1964 Helsinki Declaration and its later amendments or comparable ethical standards. The Institutional Ethics Committee approved this study (approval No. 2544), and written informed consent was obtained from patients with unruptured intracranial aneurysms or relatives as a legal representative of SAH patients.

### 4.1. Study Population

A retrospective analysis of the baseline demographic and clinical data and plasma samples collected in the pSEED was conducted in Mie University Hospital and its eight affiliated primary stroke centers in Mie prefecture in Japan from September 2013 to December 2015 (listed in Appendix A) [24,25,26]. The inclusion criteria were ≥20 years old at SAH onset; mRS scores of 0–2 before SAH onset; the detection of SAH on CT scans or MR images; ruptured saccular aneurysm diagnosed via CTA, MR angiography, or DSA; aneurysmal obliteration via microsurgical neck clipping within 48 h of SAH onset; and serial collection of plasma samples at 1–3, 4–6, 7–9 and 10–12 days post-SAH and after aneurysmal obliteration. The exclusion criteria were as follows: ruptured dissecting, traumatic, mycotic, or arteriovenous malformation-related aneurysms or SAH of unknown etiology; endovascularly treated ruptured aneurysm; severe systemic complications, including cardiac, hepatic, and/or renal dysfunction that limit DCI management; lack of imaging tests such as CTA and DSA to diagnose CVS; incomplete sample collection; diseases or conditions that may affect plasma OPN levels, such as inflammatory diseases, malignancies, and administration of immunosuppressants [10,40]; and complications such as bacterial infection and aortic dissection, which may alter the coagulation–fibrinolysis system. Regarding treatment modalities, clipping and coil embolization differ in invasiveness and influenced plasma OPN levels (Supplementary Figure S3). In addition, the number of patients treated with coil embolization was small in this cohort (cases fulfilling other criteria for this study, n = 25). Therefore, we only included SAH patients treated with aneurysmal clipping in this study.

After the diagnosis of a ruptured intracranial aneurysm, surgical clipping was performed if deemed appropriate by the attending neurosurgeons. A total of 211 consecutive SAH patients were registered, and cases with mRS scores of ≥3 before onset (n = 5), ruptured dissecting aneurysms (n = 5), aneurysmal obliteration 48 h after SAH (n = 10), ruptured aneurysm treatment other than neck clipping (n = 44), lack of imaging tests to diagnose CVS (n = 7), incomplete samples (n = 4), and diseases or conditions that may affect plasma levels of FL-OPN or OPN N-half (n = 28) were excluded. Finally, 108 SAH patients were enrolled to investigate the association of plasma OPN N-half levels with plasma FL-OPN levels and DCIn (Figure 1).

### 4.2. Clinical Variables

Baseline demographic variables were as follows: age, sex, pre-onset mRS, underlying conditions (hypertension, diabetes mellitus, and dyslipidemia), a history of cerebral infarction, smoking history, regular alcohol consumption, family history of aneurysmal SAH, preoperative WFNS grade, modified Fisher CT grade [41] at admission, acute hydrocephalus, and the site of ruptured aneurysms. Treatment-related variables included CSF drainage (ventricular, cisternal, and spinal drainage), surgery-related complications (cerebral infarction and contusion), prophylactic medications for CVS and DCI (intravenously injected fasudil hydrochloride and orally or enterally administered cilostazol), and endovascular treatments for CVS (intra-arterial injections of fasudil hydrochloride and percutaneous transluminal angioplasty). Outcome variables included DCI, angiographic CVS, DCIn, and mRS at 3 months post-SAH. The treatment and management strategies for a ruptured aneurysm, including the timing of aneurysmal obliteration, acute hydrocephalus, CVS, DCI, and other medical conditions, were determined by the on-site neurosurgeons without any limitations. Ventricular drainage was performed to manage acute hydrocephalus in all patients and to control brain swelling or increased intracranial pressure in selected patients, while cisternal drainage was performed in the basal cistern to promote the clearance of blood in the subarachnoid space during microsurgical clipping in some cases, according to the preference of treating neurosurgeons. Lumbar spinal drainage was performed when ventricular dilatation occurred postoperatively and within 14 days post-SAH.

Acute hydrocephalus was diagnosed via ventriculomegaly on CT scans at admission, when it was associated with impaired consciousness. Clipping-related complications were diagnosed on CT scans on the first post-operative day. DCI was diagnosed when otherwise unexplained neurological deterioration such as focal deficits and/or a decrease of at least 2 points on the Glasgow Coma Scale was observed and continued for more than an hour [2]. Angiographic CVS was diagnosed when CTA or DSA revealed a reduction of more than 50% in the vessel diameter of major intracranial cerebral arteries compared to the baseline, regardless of the presence of the symptoms. DCIn was defined as a newly identified cerebral infarction on CT scans or MR images that was not recognized on the initial postoperative scans or images. Good and poor outcomes were defined as mRS scores of 0–2 and 3–6 at 3 months post-SAH, respectively. The attending neurosurgeons at each center diagnosed these events, and the organizing committee qualified them.

### 4.3. Measurement of Plasma OPN Levels

Blood samples were serially collected with minimal stasis from peripheral veins early in the morning at 1–3, 4–6, 7–9, and 10–12 days post-SAH and after aneurysmal obliteration. They were immediately centrifuged at 3000 rpm for 5 min to separate cellular materials from the supernatant plasma, which was stored at −78 °C until analysis. The FL-OPN and OPN N-half levels in stored plasma samples were measured using a commercially available enzyme-linked immunosorbent assay kit for human OPN (27158; IBL, Fujioka, Japan) and human OPN N-half (27258; IBL, Fujioka, Japan), respectively, by an experienced technician who was blinded to the clinical data.

As a control, plasma samples were collected from 8 patients with unruptured intracranial cerebral aneurysms (5 males and 3 females, *p* = 0.108 versus SAH patients, Fisher’s exact test; 69.0 (66.5–71.0) years old, *p* = 0.423 versus SAH patients, Mann–Whitney U test) who had no comorbidities influencing plasma OPN levels, such as inflammatory diseases, malignancies, and aortic dissection, and they were used as reference to measure plasma FL-OPN and OPN N-half levels in the same manner.

### 4.4. Statistical Analysis

All statistical analyses were performed with SPSS Statistics version 29.0.2.0 (IBM, Armonk, NY, USA). Categorical variables were expressed as numbers (percentages) and compared between the two groups using chi-squared or Fisher’s exact tests, as appropriate [42]. Outliers were assessed using the Smirnov–Grubbs test at a significance level of 0.05, and data were extracted for analysis. Normally distributed continuous variables in the Shapiro–Wilk test were expressed as means ± standard deviations and compared between the two groups using an unpaired *t*-test, while non-normally distributed continuous variables were expressed as medians and interquartile ranges and compared between the two groups using the Mann–Whitney U test. The values on graphs were indicated as a mean ± standard error of the mean.

ROC curves and the AUC were analyzed to determine the optimal cut-off values of plasma concentrations of FL-OPN and OPN N-half to detect DCIn occurrence using the Youden index. The sensitivity and specificity were calculated for these cut-off values to differentiate patients with DCIn from those without. Multivariate logistic regression analyses with the presence or absence of DCIn as the dependent variable were performed using a forward stepwise method, whereas any variables with *p* values of <0.05 were used in univariate analyses, although only the one with the smallest *p* value among the intercorrelated clinical variables and a Spearman’s correlation coefficient (r) > 0.3 was used as a candidate variable. The aOR and the 95% CI were calculated, and the independence of variables was assessed using the likelihood ratio test on reduced models. A *p* value < 0.05 was considered significant.

## Figures and Tables

**Table 1:** Baseline clinical variables in patients with and without delayed cerebral infarction (DCIn).

Variables	Total (n = 108)	DCIn (n = 23)	No DCIn (n = 85)	*p* Value
Age, mean ± SD (years)	63.6 ± 14.0	68.6 ± 12.2	62.3 ± 14.2	0.054 ^a^
Age ≥ 75 years old	23 (21.3%)	7 (30.4%)	16 (18.8%)	0.256 ^b^
Sex, female	76 (70.4%)	17 (73.9%)	59 (69.4%)	0.871 ^c^
Underlying condition				
Cerebral infarction	6 (5.6%)	1 (4.3%)	5 (5.9%)	1.000 ^b^
Hypertension	43 (39.8%)	9 (39.1%)	34 (40.0%)	1.000 ^c^
Dyslipidemia	12 (11.1%)	1 (4.3%)	11 (12.9%)	0.455 ^b^
Diabetes mellitus	6 (5.6%)	0 (0.0%)	6 (7.1%)	0.388 ^b^
Family history of SAH	11 (10.2%)	0 (0.0%)	11 (12.9%)	0.116 ^b^
Smoking history	25 (23.1%)	6 (26.1%)	19 (22.4%)	0.992 ^c^
Regular alcohol	18 (16.7%)	4 (17.4%)	14 (16.5%)	1.000 ^b^
mRS before onset				
0	98 (90.7%)	21 (91.3%)	77 (90.6%)	1.000 ^b^
1	9 (8.3%)	1 (4.3%)	8 (9.4%)	0.681 ^b^
2	1 (0.9%)	1 (4.3%)	0 (0.0%)	0.213 ^b^
Preoperative WFNS grade				
I	28 (25.9%)	3 (13.0%)	25 (29.4%)	0.187 ^c^
II	31 (28.7%)	7 (30.4%)	24 (28.2%)	1.000 ^c^
III	10 (9.3%)	3 (13.0%)	7 (8.2%)	0.441 ^b^
IV	20 (18.5%)	4 (17.4%)	16 (18.8%)	1.000 ^b^
V	19 (17.6%)	6 (26.1%)	13 (15.3%)	0.231 ^b^
IV–V	39 (36.1%)	10 (43.5%)	29 (34.1%)	0.559 ^c^
Modified Fisher grade				
1	14 (13.0%)	0 (0.0%)	14 (16.5%)	0.038 ^b^
2	6 (5.6%)	0 (0.0%)	6 (7.1%)	0.338 ^b^
3	60 (55.6%)	16 (69.6%)	44 (51.8%)	0.198 ^c^
4	28 (25.9%)	7 (30.4%)	21 (24.7%)	0.773 ^c^
3–4	88 (81.5%)	23 (100%)	65 (76.4%)	0.006 ^b^
Acute hydrocephalus	41 (38.0%)	6 (26.1%)	35 (41.2%)	0.280 ^c^
Ruptured aneurysm location				
ICA	43 (39.8%)	8 (34.8%)	35 (41.2%)	0.752 ^c^
MCA	21 (19.4%)	8 (34.8%)	13 (15.3%)	0.070 ^b^
AcomA	30 (27.8%)	7 (30.4%)	23 (27.1%)	0.954 ^c^
ACA	8 (7.4%)	0 (0.0%)	8 (9.4%)	0.198 ^b^
VA-BA	6 (5.6%)	0 (0.0%)	6 (7.1%)	0.338 ^b^

Data are expressed as number of patients (%) unless otherwise specified. *p* values are determined using an ^a^ unpaired *t*-test, ^b^ Fisher’s exact test, or ^c^ Pearson’s chi-squared test. ACA, anterior cerebral artery; AcomA, anterior communicating artery; ICA, internal carotid artery; MCA, middle cerebral artery; mRS, modified Rankin Scale; SAH, subarachnoid hemorrhage; SD, standard deviation; VA-BA, vertebral artery and basilar artery; WFNS, World Federation of Neurological Surgeons.

**Table 2:** Treatment-related variables in patients with and without delayed cerebral infarction (DCIn).

Variables	Total (n = 108)	DCIn (n = 23)	No DCIn (n = 85)	*p* Value
Treatment-related complication	21 (19.4%)	6 (26.1%)	15 (17.6%)	0.381 ^a^
Cerebral infarction	17 (15.7%)	4 (17.4%)	13 (15.3%)	0.756 ^a^
Cerebral contusion	4 (3.7%)	2 (8.7%)	2 (2.4%)	0.198 ^a^
CSF drainage	47 (43.5%)	10 (43.5%)	37 (43.5%)	1.000 ^b^
Ventricular drainage	35 (32.4%)	5 (21.7%)	30 (35.3%)	0.327 ^b^
Cisternal drainage	23 (21.3%)	4 (17.4%)	19 (22.4%)	0.777 ^a^
Spinal drainage	10 (9.3%)	4 (17.4%)	6 (7.1%)	0.215 ^a^
Prophylaxis for CVS or DCI				
i.v. Fasudil hydrochloride	107 (99.1%)	23 (100%)	84 (98.8%)	1.000 ^a^
Oral or enteral cilostazol	87 (80.6%)	17 (73.9%)	70 (82.4%)	0.381 ^a^
Angiographic CVS	40 (37.0%)	16 (69.6%)	24 (28.2%)	<0.001 ^b^
DCI	18 (16.7%)	12 (52.2%)	6 (7.1%)	<0.001 ^a^
Endovascular CVS treatment				
i.a. Fasudil hydrochloride	9 (8.3%)	5 (21.7%)	4 (4.7%)	0.020 ^a^
3-month mRS				
0	35 (32.4%)	4 (17.4%)	31 (36.5%)	0.138 ^b^
1	16 (14.8%)	1 (4.3%)	15 (17.6%)	0.184 ^a^
2	13 (12.0%)	2 (8.7%)	11 (12.9%)	0.731 ^a^
3	15 (13.9%)	5 (21.7%)	10 (11.8%)	0.305 ^a^
4	9 (8.3%)	2 (8.7%)	7 (8.2%)	1.000 ^a^
5	15 (13.9%)	5 (21.7%)	10 (11.8%)	0.305 ^a^
6	5 (4.6%)	4 (17.4%)	1 (1.2%)	0.007 ^a^
0–2	64 (59.3%)	7 (30.4%)	57 (67.1%)	0.003 ^b^

Data are expressed as number of patients (%) unless otherwise specified. *p* values are determined using ^a^ Fisher’s exact test or ^b^ Pearson’s chi-squared test. CSF, cerebrospinal fluid; CVS, cerebral vasospasm; DCI, delayed cerebral ischemia; i.a., intra-arterial; i.v., intravenous; mRS, modified Rankin Scale.

**Table 3:** Multivariate logistic regression analysis with delayed cerebral infarction (DCIn) as the dependent variable using plasma levels of N-terminal fragments of osteopontin cleaved by thrombin (OPN N-half) with a value of ≥70.42 pmol/L at days 10–12 and significant variables related to DCIn incidence on univariate analysis.

Variables	aOR	95% CI	*p* Value
DCI	10.49	2.89–38.12	<0.001
Plasma OPN N-half ≥ 70.42 pmol/L at days 10–12	5.65	1.68–18.97	0.005
Modified Fisher grades 3–4			0.998

aOR, adjusted odds ratio; CI, confidence interval; DCI, delayed cerebral ischemia.

## Data Availability

The data from this study will be made available to qualified investigators upon reasonable request.

## Supplementary Materials

The following supporting information can be downloaded at: https://www.mdpi.com/article/10.3390/ijms26062781/s1.

## Abbreviations

aOR, adjusted odds ratio; AUC, area under the curve; CI, confidence interval; CSF, cerebrospinal fluid; CT, computed tomography; CTA, computed tomography angiography; CVS, cerebral artery vasospasm; DCI, delayed cerebral ischemia; DCIn, delayed cerebral infarction; DSA, digital subtraction angiography; FL-OPN, full-length osteopontin; MR, magnetic resonance; mRS, modified Rankin Scale; OPN, osteopontin; OPN N-half, N-terminal half of osteopontin; pSEED, Prospective Registry for Searching Mediators of Neurovascular Events After Aneurysmal Subarachnoid Hemorrhage; RGD, Arg-Gly-Asp; ROC, receiver-operating characteristic; SAH, subarachnoid hemorrhage; SVVYGLR, Ser-Val-Val-Tyr-Gly-Leu-Arg; TGF, transforming growth factor; WFNS, World Federation of Neurological Surgeons.

## Appendix A

Prospective Registry for Searching Mediators of Neurovascular Events After Aneurysmal Subarachnoid Hemorrhage (pSEED) Group: Department of Neurosurgery, Kuwana City Medical Center, Kuwana (Hiroshi Sakaida, Yasuyuki Umeda, Katsuhiro Tanaka, and Yoichi Miura); Mie Prefectural General Medical Center, Yokkaichi (Yusuke Kamei, and Takanori Sano); Suzuka Kaisei Hospital, Suzuka (Tomohiro Araki, Masato Shiba, and Naoki Ichikawa); Suzuka Chuo General Hospital, Suzuka (Shigetoshi Shimizu, Takuro Tsuchiya, and Reona Asada); Mie University Graduate School of Medicine, Tsu (Hidenori Suzuki, Naoki Toma, Ryuta Yasuda, Fumihiro Kawakita, Yoshinari Nakatsuka, Hirofumi Nishikawa, Hideki Kanamaru, and Yotaro Kitano); Mie Chuo Medical Center, Tsu (Fujimaro Ishida, Keiji Fukazawa, and Satoru Tanioka); Matsusaka Chuo General Hospital, Matsusaka (Kazuhiko Tsuda, and Yu Sato); Saiseikai Matsusaka General Hospital, Matsusaka (Hiroto Murata, and Kazuhide Hamada); Ise Red Cross Hospital, Ise (Fumitaka Miya, Hiroshi Tanemura, and Masashi Fujimoto).
