# Supplementary material for: Increased Plasma Levels of Thrombin-Cleaved Osteopontin in Patients with Delayed Cerebral Infarction After Aneurysmal Subarachnoid Hemorrhage
Source: Int J Mol Sci. 2025 Mar 19;26(6):2781. doi: 10.3390/ijms26062781 (PMC11943441; doi:10.3390/ijms26062781)
Supplement: Supplementary file 1 [file ijms-26-02781-s001.zip › ijms-3481092-supplementary.pdf]

Supplementary Figures and Figure Legends

Increased Plasma Levels of Thrombin-Cleaved Osteopontin in Patients with Delayed Cerebral Infarction After Aneurysmal Subarachnoid Hemorrhage

Kazuaki Aoki, Fumihiro Kawakita, Koichi Hakozaki, Hideki Kanamaru, Reona Asada, Hidenori Suzuki and pSEED Group

Address correspondence to: Hidenori Suzuki, M.D., Ph.D.
Department of Neurosurgery, Mie University Graduate School of Medicine,
2-174 Edobashi, Tsu, Mie 514-8507, Japan
E-mail: suzuki02@med.mie-u.ac.jp

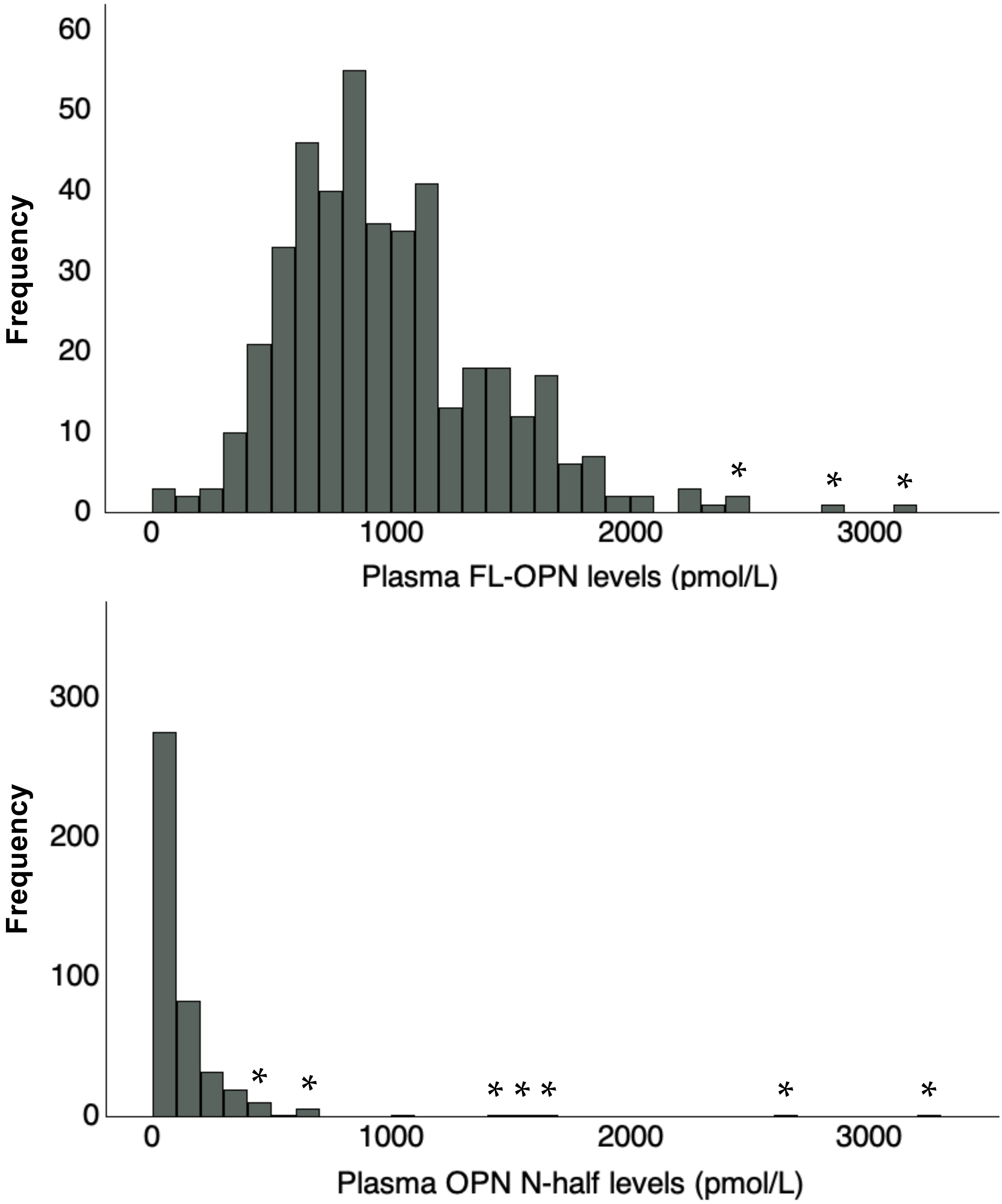

Figure S1. The histogram of plasma levels of full-length osteopontin (FL-OPN; *upper*) and N-terminal fragments of osteopontin cleaved by thrombin (OPN N-half; *lower*).
*Outliers tested with the Smirnoff-Grubbs test.

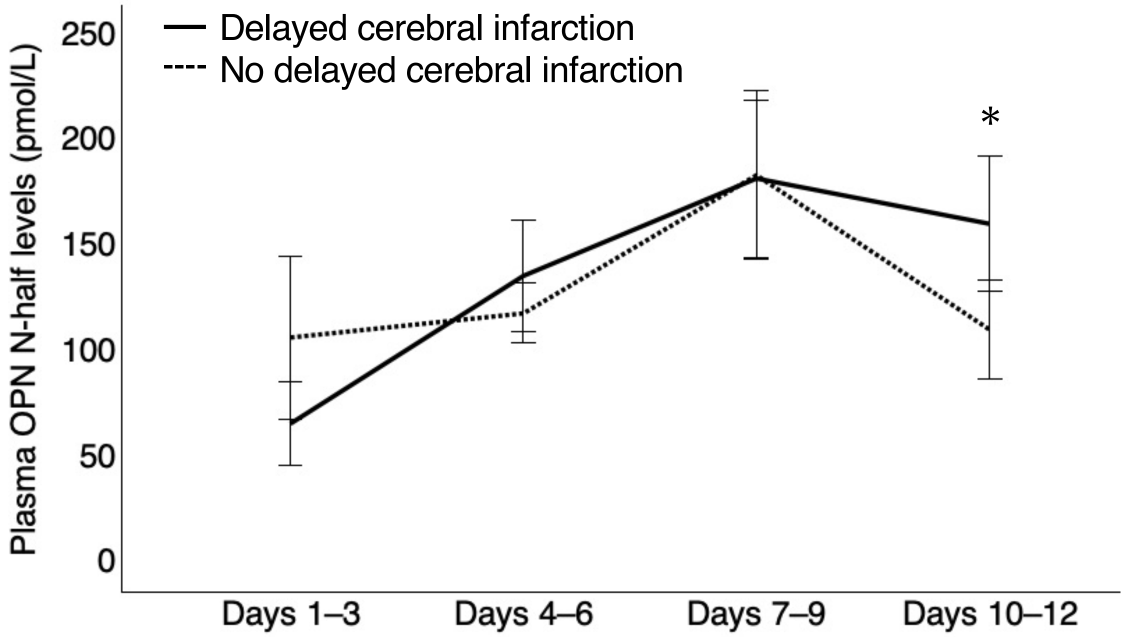

Figure S2. Comparison of plasma levels of N-terminal fragments of osteopontin cleaved by thrombin (OPN N-half) between patients with and without delayed cerebral infarction (n=23 and 85, respectively) after subarachnoid hemorrhage.

Data are expressed as a mean ± standard error of the mean without exclusion of outliers.

*p = 0.001 versus no delayed cerebral infarction, Mann–Whitney U test.

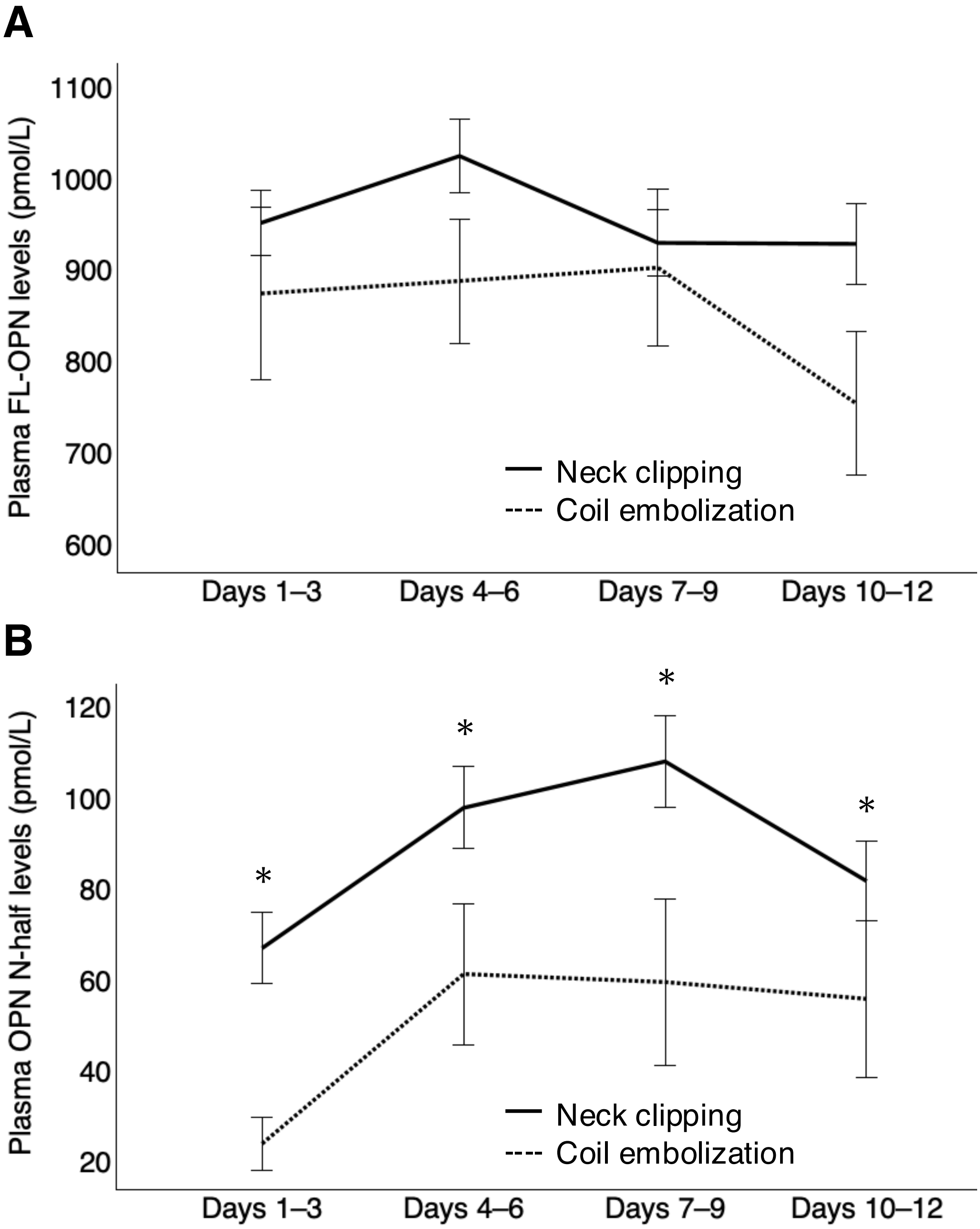

Figure S3. Comparison of plasma levels of full-length osteopontin (FL-OPN; **A**) and N-terminal fragments of osteopontin cleaved by thrombin (OPN N-half; **B**) between patients with aneurysmal subarachnoid hemorrhage treated by neck clipping (n=108) and coil embolization (n=25).

Data are expressed as a mean ± standard error of the mean after exclusion of outliers.

*$p < 0.05$ versus coil embolization, Mann–Whitney U test.
